# Impression management in daily life: an experience sampling test for the expression of impression management as interpersonally oriented self-control

**DOI:** 10.3389/fpsyg.2023.1198891

**Published:** 2023-08-28

**Authors:** Liad Uziel, Tomer Schmidt-Barad

**Affiliations:** ^1^Department of Psychology, Bar-Ilan University, Ramat-Gan, Israel; ^2^Department of Behavioral Science, Peres Academic Center, Rehovot, Israel

**Keywords:** impression management, self-control, social presence, interpersonally oriented self-control, aloneness, agreeableness, self-deception

## Abstract

Impression management (IM) scales (often called lie or social desirability scales) have long been applied as validity scales in assessment processes. Recent developments have indicated that these scales measure a substantive personality predisposition and not response bias, but the nature of the disposition is disputable. According to the ‘interpersonally oriented self-control’ approach, IM is associated with high self-control exerted mainly in public social contexts to facilitate adaptation. Supported in laboratory settings, this approach has not been tested in real-life dynamics. In the present experience sampling study, participants reported 3 times a day (10 days) about their social condition (alone/'with others’) and their level of self-control. Results revealed that IM was associated with stronger self-control when with other people than when alone. Comparable reactions to public social context were not found for self-deception enhancement, trait self-control, or agreeableness, marking this a unique aspect of IM. The findings further stress the need to reconsider the use of IM scales for validity purposes in assessment processes.

## Introduction

Scales of impression management—also called validity, social desirability, or lie scales—have been an integral part of personality assessment since its early days (see Uziel, 2010b, for a review). Researchers administering personality questionnaires quickly realized that the validity of self-reports is often compromised because (some) respondents bias their responses (Ellis, 1946). Soon after, attempts to control for response biases gained momentum. The use of social desirability scales was popularized following work by Edwards (1953, 1957) who devised an individual difference tool constructed from items of the Minnesota Multiphasic Personality Inventory (MMPI). Following this, more than a dozen scales were created to measure social desirability (Wiggins, 1964), with the most popular one being the Marlowe-Crowne Social Desirability Scale (MCSDS; Crowne and Marlowe, 1960, Crowne and Marlowe, 1964).

Additional developments uncovered limitations of the MCSDS and indicated that social desirability is a two-dimensional construct. Specifically, following distinctions made by Wiggins (1964) and Sackeim and Gur (1978), Paulhus (1984, 2002) described a model for social desirability that differentiates between impression management (IM) and self-deception enhancement (SDE) and introduced the Balanced Inventory of Desirable Responding (BIDR). IM is said to reflect a habitual and conscious tendency to portray an overly positive self-portrait, whereas SDE reflects an unconscious self-serving bias. It has generally been recommended to control for IM in scale development and administration because biases associated with SDE persist beyond the setting of responding to questionnaires (Paulhus, 1984).

The application of social desirability (specifically, IM) scales to control for response bias has become ubiquitous in research and practice across diverse fields, such as personality, social, clinical, and organizational psychology (e.g., Goffin and Christiansen, 2003; Logan et al., 2008; Uziel, 2010b; Perinelli and Gremigni, 2016; Barry et al., 2017; Maltby et al., 2019). Moreover, individuals with high scores on IM scales were considered to experience enduring psychological maladjustment. Specifically, Crowne and Marlowe (1964; see also Crowne 1979) have suggested that a high IM score reflects a defense mechanism that shields individuals with vulnerable (i.e., low and insecure) self-esteem. This vulnerability is associated with social anxiety, defensive and awkward social manners, and poor adjustment (see also Weinberger et al., 1979). Thus, interest in IM scales stemmed from the dire real-life implications that have been attributed to high scorers in addition to their role as validity tools.

Over the years, evidence has accumulated suggesting that IM scales are ineffective as measures of response bias and that they contain more (personality) substance than (response) style (e.g., McCrae and Costa, 1983; Ones et al., 1996; Uziel, 2010b; MacCann et al., 2012; Müller and Moshagen, 2019).^1^ Evidence along this line has shown, for example, that IM scales fail to moderate or suppress criterion-related validities of personality traits in occupational contexts (Li and Bagger, 2006), that they fail to moderate self-other agreement on personality traits (Borkenau and Ostendorf, 1992; Uziel, 2014; Müller and Moshagen, 2019), that knowledgeable others attribute reliability and trustworthiness to individuals with high IM scores (De Vries et al., 2014), as do direct behavioral tests of cheating (Zettler et al., 2015). More so, like other personality traits—but unlike a contextual response set—scores on the IM scale show consistency across situations and over time (Lönnqvist et al., 2007), and they reliably predict real-life behaviors (Uziel, 2010b).

However, a persistent question concerns the substance that IM scales measure. Various studies have identified different correlates of IM. For example, McCrae and Costa (1983) have noted that IM is associated with emotional stability and warmth, and Ones et al. (1996) have concluded (in a meta-analysis) that IM is associated with emotional stability, agreeableness, and conscientiousness. More recent approaches have refined the associations, pinpointing elements of interpersonal orientation and social responsiveness. Bou Malham and Saucier (2016) have associated IM with cultural normativity, representing both knowledge and successful application of cultural values by high-IM individuals (see also Elliot et al., 2018). De Vries et al. (2014) have associated IM with honesty-humility, suggesting that high scorers are sincere, fair, and modest (see also Paulhus, 2002).

These approaches are informative in highlighting the correlates of IM. However, there is a need to accommodate their diverse findings under an overarching model. More so, by and large, they are based on cross-sectional data. Arguably, an exhaustive account of IM and its relation to social behavior would incorporate the social context and the dynamics in the expression of this trait in varying settings (Mischel and Shoda, 1995; Uziel, 2010b).

### Interpersonally oriented self-control

In recent years, an approach to IM has been proposed that seeks to incorporate the dynamics of social responsiveness under the umbrella of a general mechanism. According to the *interpersonally oriented self-control* (IOSC) approach, individuals high on IM are characterized by high self-control expressed mainly in social contexts (Uziel, 2010b, 2014). That is, high-IM individuals are highly responsive to social contexts, meaning that they are inclined to modify their behavior in response to others’ presence. In addition, they can adjust their self-regulatory efforts to meet the demands of social situations, reflecting a *responsivity-ability duo*.

High-IM promotes social adjustment through a controlled and collected behavioral style. In the terminology of the interpersonal circumplex model (Trapnell and Wiggins, 1990), high-IM individuals are portrayed as high on affiliation and low on dominance. In the transition from private to public contexts, strong affiliation motivates behavioral adjustments and low dominance necessitates the adjustments. High self-control facilitates their success in making these adjustments and is thus a central component of the model (Uziel and Baumeister, 2012). Ironically, individuals high on IM often do not leave a very strong first impression on others. Their collected manner makes their social qualities appreciated gradually, through extended acquaintanceships (Paulhus, 1998). From an evolutionary perspective, their manner fits a ‘slow life history’ strategy, which values long-term goals, high-quality ends, and that builds on strong inhibitory control and inclination toward cooperation (Ellis et al., 2009).

Evidence in support of the IOSC approach to IM comes from diverse sources (see Uziel, 2010b, for a review). As described above, in self-reports and informant reports high-IM is associated with traits incorporating sensitive social attunement and with trait self-control (Tangney et al., 2004; Uziel, 2014). Real-life outcomes also imply that IM is associated with controlled (social) behavior. For example, IM is positively associated with the likelihood of getting married and staying married (Harker and Keltner, 2001), religiosity (Gillings and Joseph, 1996), overall social integration (Watson et al., 1995; Guo et al., 2019), and it is negatively correlated with drug abuse and alcoholism (Bradburn et al., 1979).

Experimental evidence supports the responsiveness aspect of high-IM individuals’ social behavior. High-IM individuals were found to display increased physiological reactions in response to social presence (Kline et al., 2002; Pauls et al., 2005), and behavioral evidence has shown that in response to social presence (vs. alone) high-IM individuals display greater self-control, more creativity, and overall improved performance on demanding tasks (Uziel, 2010a; Uziel and Baumeister, 2012). Moreover, when faced with social stress, high IM was shown to act as a buffer, helping individuals respond adaptively to rejection (Blackhart et al., 2007; Guo et al., 2019).

In sum, accumulated evidence indicates that IM is associated with interpersonally oriented self-control, based on self and informant reports, on (distal) real-life outcomes, and experimentally induced behavioral responses to the mere presence of others and social stress. Yet, evidence in support of the responsiveness of high-IM individuals to social presence (vs. alone) is still limited to laboratory manipulations, which are often brief and structured. Therefore, evidence for a (self-controlled) reaction to social conditions under real-life settings could add much-needed information about the substantive nature of this trait.

### The present research

The present study was set to explore the response of individuals high on IM (and related constructs) to public social contexts (vs. alone) in terms of the level of momentary self-control. We sought to do so in people’s natural environment. Specifically, using an experience sampling method, participants were approached in their everyday life and were asked to report (3 times a day for 10 days) about their current social condition (alone/with other people), and their momentary level of self-control. We predicted that individuals high (vs. low) on IM will respond to being with others with an increase in their self-control, and we expected this to be a unique aspect of IM when compared with related constructs.

### Discriminant validity

The construct validity of IM may gain by differentiating it from related constructs. To do so, we compared the response pattern associated with IM to three constructs: Self-deception enhancement, trait self-control, and agreeableness. We briefly describe their respective differences in the context of the present research.

### Self-deception enhancement

In the literature on response bias, self-deception enhancement (SDE) has been considered another expression of social desirability, reflecting a non-conscious bias associated with narcissistic exaggeration of one’s abilities (Paulhus, 1984, 2002). Despite a moderate correlation with IM, evidence indicates that SDE taps more style than substance, including unsubstantiated self-attributed high self-control (Sackeim and Gur, 1979; Uziel, 2014). Importantly, given the inward-directedness nature of the bias, self-deceiving individuals are considered insensitive to changes in social contexts (Paulhus, 1984). Therefore, they are expected to report (albeit not necessarily show) high levels of self-control in both settings (alone and with other people).

### Trait self-control

Self-control is an important component of IM, but what characterizes individuals high on trait self-control (TSC) is their internalized self-regulatory capacity, which serves them across contexts (Tangney et al., 2004). High-TSC individuals are considered highly effective managers of internal conflicts, impulses, and behavioral regulation (Metcalfe and Mischel, 1999). Sure enough, these capabilities facilitate social integration (Baumeister, 2005), yet there is little evidence that directly connects TSC with reactivity in public social contexts. Therefore, high-TSC individuals are expected to report higher levels of self-control than low-TSC individuals, yet the current state of knowledge does not suggest that high-TSC individuals respond to public social contexts with a greater increase in self-control than low-TSC individuals.

### Agreeableness

Agreeableness reflects individual differences in the motivation to maintain positive relations with others. Individuals high on agreeableness are emphatic, altruistic, and place the interests of others at the forefront of their thoughts (Graziano and Eisenberg, 1997). Despite the apparent similarity with IM, the two constructs are only moderately correlated (~0.25; Graziano and Tobin, 2002). A possible factor accounting for the modest overlap is that altruistic motives are not a strong component in IM (Uziel, 201b). IM is focused on changes in the *self* to accommodate social environments, not so much on others’ welfare. Agreeableness is often associated with effortful control, the ability to suppress dominant responses (and an important component of self-control; Rothbart and Ahadi, 1994). And yet, among agreeable individuals, this ability is expressed in prosocial behavior (Graziano et al., 2007). That is, agreeable individuals are responsive to other people’s distress. However, their general responsiveness to others’ presence in common daily activities (i.e., non-distress related) has not been documented. Thus, individuals high on agreeableness are expected to report higher self-control than individuals low on agreeableness, but this difference is not expected to increase substantially in the presence of other people (vs. alone).

### Statistical power and open science declaration

A Monte-Carlo simulation (Arend and Schäfer, 2019) advised that 150 participants (each providing 30 ratings) yield 80% power for detecting small (cross-level interaction) effects (*r* ≥ 0.24, *R*^2^ ≥ 0.06). Our final sample (*N* = 154) followed this benchmark. Materials, data, and code for the analyses are posted on the Open Science Framework: https://osf.io/kqy5t/.

## Materials and methods

### Participants and procedure

Participants were students recruited for an intensive longitudinal study about social experiences and psychological states. One hundred fifty-five participants started the study. One participant was dropped after systematically failing to complete episodic reports, setting the final sample at *N* = 154 (Females = 125; Males = 29; *M*_age_ = 23.92, *SD_age_* = 3.12). The project had multiple goals, some unrelated to the present question (the current variables are not part of other publications stemming from this project, except for the reference to the current social condition; e.g., Uziel and Schmidt-Barad, 2022).

At a start-up session, participants completed personality and demographic questionnaires. Episodic data were collected by prompting participants (with text messages) to report 3 times a day (morning, noon, and evening) for 10 consecutive days (excluding weekends). Episodic reports started by asking about the current social condition (alone/with other people), followed by questions about the current mental state, including the focus of the present research—level of self-control. Completion of each episodic report lasted approximately 2 min (*Median* = 117 s; 5% trimmed *Mean* = 138 s).

## Materials

### Trait questionnaires

#### Impression management (IM) and Self-deception enhancement (SDE)

Participants completed the Balanced Inventory of Desirable Responding (BIDR-6; Paulhus, 1991), which consists of 40 items measuring IM (20 items, e.g., “I never swear”; α = 0.81) and SDE (20 items, e.g., “My first impressions of people usually turn out to be right”; α = 0.75). Participants rated their level of agreement with each statement using a 7-point Likert-type scale anchored with 1-*not true* and 7-*very true*.

#### Trait self-control

TSC was measured with the brief (13-item) version of the Trait Self-Control Scale (Tangney et al., 2004; e.g., “I refuse things that are bad for me”; α = 0.87), which is the most widely applied self-control scale in recent years (De Ridder et al., 2012). Participants rated the extent to which each item reflects who they *typically* are using a 5-point Likert-type scale anchored with 1-*not at all* and 5-*very much*.

#### Agreeableness

Participants completed the Mini-Markers (Saucier, 1994). The Mini-Markers consist of 40 adjectives, eight of which measure Agreeableness (e.g., “Warm,” “Kind”; α = 0.67). Participants rated the extent to which each item accurately describes them using a 5-point Likert-type scale ranging from 1-*completely inaccurate* to 5-*completely accurate*.

### Episodic reports

#### Social condition

Participants reported with one item whether they are *currently* alone or with other people. Alone was defined as being physically alone while not actively communicating with other people. ‘With others’ was defined as being in the same space with others, and/or actively communicating with other people.

#### Episodic self-control

Participants marked how well each of 2 items (“I am good at resisting temptation,” “I’m not easily discouraged”) describes their *current state* on a 1*-strongly disagree* to 5*-strongly agree* scale. In having 2 items we followed recommendations concerning the length of episodic reports in experience-sampling research (Csikszentmihalyi and Larson, 2014). We used these specific items (originally from the Trait Self-Control Scale; Tangney et al., 2004) because they applied to episodic experiences while still general enough to a range of contexts. To calculate the reliability of the scale we differentiated the between-subject and within-subject levels (Nezlek, 2017; Yang et al., 2022), using the R shiny web application https://psychmethods.shinyapps.io/withinpersonresearch/ (Yang et al., 2022). The between-subject reliability was α = 0.70. The within-subject reliability was α = 0.49, which is considered fair for this type of data (i.e., a 2-item episodic scale) according to current best-practice recommendations (e.g., Shrout, 1998; Nezlek, 2017).

## Results

### Analysis overview and descriptive statistics

Given the clustered nature of the data (episodic reports nested within individuals), results were analyzed by multilevel modeling (with SPSS Mixed procedure and jamovi’s GAMLj module for additional computations, such as simple slopes; Gallucci, 2019; The jamovi project, 2021). Following Bolger et al.’s (2003) recommendation, we controlled for *time* in the main analyses (i.e., the specific sampling point).^2^

The 154 participants provided 4,264 momentary reports, yielding a mean of 27.71 (*SD* = 5.98) reports per participant, reflecting a high response rate (92.29%). Across all episodes, participants were alone 37.1% of the time and with other people 62.9% of the time.

To gain an overview of the relations between the variables in the study, Table 1 presents Means, *SD*s, and correlations (note that for this analysis the Level-1 variables—social condition and episodic self-control—were averaged across episodes within individuals). There are several noteworthy associations in this table. First, IM was positively correlated with TSC, SDE, and agreeableness, which were positively intercorrelated. Second, consistent with past findings (e.g., Uziel, 2014), SDE had the strongest correlation with trait self-control. Third, all trait constructs were positively correlated with episodic self-control. Interestingly, the correlation between TSC and episodic self-control was moderate (*r* = 0.47), supporting inferences that trait and state measures of the same construct often tap (somewhat) different processes (e.g., Fleeson, 2001; Hofmann et al., 2012; Hudson et al., 2020). Forth, also positive, albeit weakly, was the correlation between being more often with other people and (averaged) episodic self-control, implying that people who reported being more often with others also used more self-control (Erber et al., 1996). Last, none of the traits were associated with being more often with other people.

**Table 1:** Means and correlations of the variables in the study.

Variable	M (SD)	2	3	4	5	6
1. Social condition^a^	0.63 (0.17)	0.04	0.14	0.11	0.07	0.18*
2. IM	4.37 (0.86)	–	0.39**	0.46**	0.30***	0.27**
3. SDE	4.17 (0.68)		–	0.54**	0.24**	0.51***
4. TSC	3.16 (0.66)			–	0.23**	0.47***
5. Agreeableness	4.16 (0.46)				–	0.31***
6. Episodic self-control	3.48 (0.62)					–

*N* = 154.

IM, Impression management, SDE, Self-deception enhancement, TSC, Trait self-control.

a = 0 = Alone, 1 = With other people.

* = *p* < 0.05, ** = *p* < 0.01, *** = *p* < 0.001.

These correlations are of interest at the aggregated (i.e., between-subject) level. The following analyses address our focal interest more directly by considering the dynamics of social behavior within individuals over time.

### Main analysis

The main goal of the study was to test whether individuals high on IM respond to social presence with an increase in their level of self-control and to compare their response to those associated with related traits. First, we tested an unconditional model, which revealed that 60% (*ICC* = 0.60) of the variance in episodic self-control could be attributed to interindividual differences, and the remainder to intraindividual dynamics (*p*s < 0.001). Therefore, we proceeded with multilevel modeling which considers both sources of variance.

**Figure 1:**
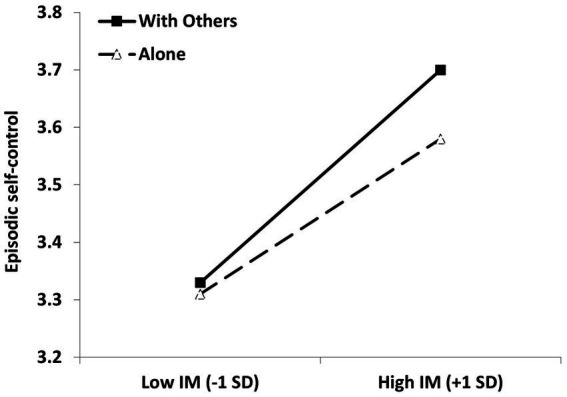
*Interaction between IM and momentary social condition in predicting episodic self-control.* IM, Impression management.

The main analysis involved a cross-level interaction between IM (Level-2; grand-mean centered) and current social condition (Level-1; dummy coded with 0 = alone and 1 = with others), allowing the intercepts and slopes to vary randomly across individuals. The results are summarized in Table 2 and visualized in Figure 1. As seen in Table 2 (top panel), IM was significantly positively associated with episodic self-control. The social condition was also a significant predictor—participants reported more self-control in the presence of other people. Importantly, there was a significant interaction between IM and social condition. Probing the interaction (Figure 1) revealed that, as predicted, higher IM was associated with a stronger effect on episodic self-control while being with other people, *b* = 0.22, *SE* = 0.06, 95% CI [0.11, 0.33], *t* (152) = 3.87, *p* < 0.001, η^2^_p_ = 0.090, than while being alone, *b* = 0.16, *SE* = 0.06, 95% CI [0.05, 0.28], *t* (150) = 2.76, *p* = 0.007, η^2^_p_ = 0.048. Probing the interaction with the alternative emphasis revealed that high-IM (+1*SD*) individuals experienced more self-control with other people than alone, *b* = 0.11, *SE* = 0.03, 95% CI [0.06, 0.17], *t* (143) = 3.95, *p* < 0.001, η^2^_p_ = 0.098. Low-IM (-1*SD*) individuals experienced the same level of self-control in both conditions, *b* = 0.02, *SE* = 0.03, 95% CI [−0.04, 0.08], *t*(144) = 0.65, *p* = 0.516, η^2^_p_ = 0.003.

**Table 2:** Multilevel modeling analyses for predicting episodic self-control by personality traits (IM, SDE, TSC, and Agreeableness) and momentary social condition.

Parameter	*B*	*SE*	*95% CI*	*t*	*p*
Impression management (IM)					
Intercept	3.44	0.05	[3.35, 3.54]	68.70	< 0.001
Time	0.005	0.001	[0.003, 0.007]	5.46	< 0.001
IM	0.16	0.06	[0.05, 0.28]	2.76	0.007
Social condition^a^	0.07	0.02	[0.03, 0.11]	3.25	0.001
IM*Social condition	0.06	0.02	[0.01, 0.10]	2.35	0.020
Self-Deception enhancement (SDE)					
Intercept	3.44	0.05	[3.36, 3.53]	76.54	< 0.001
Time	0.005	0.001	[0.003, 0.007]	5.36	< 0.001
SDE	0.45	0.07	[0.32, 0.58]	6.85	< 0.001
Social condition^a^	0.07	0.02	[0.03, 0.11]	3.19	0.002
SDE*Social condition	0.01	0.03	[−0.05, 0.07]	0.37	0.709
Trait Self-Control (TSC)					
Intercept	3.44	0.05	[3.35, 3.53]	74.73	< 0.001
Time	0.005	0.001	[0.003, 0.007]	5.41	< 0.001
TSC	0.42	0.07	[0.28, 0.56]	6.02	< 0.001
Social condition^a^	0.07	0.02	[0.03, 0.11]	3.20	0.002
TSC*Social condition	0.02	0.03	[−0.04, 0.09]	0.73	0.465
Agreeableness					
Intercept	3.43	0.05	[3.34, 3.53]	70.50	< 0.001
Time	0.005	0.001	[0.003, 0.007]	5.42	< 0.001
Agreeableness	0.42	0.11	[0.21, 0.63]	3.99	< 0.001
Social condition^a^	0.07	0.02	[0.02, 0.11]	3.15	0.002
Agreeableness*Social condition	0.02	0.05	[−0.07, 0.11]	0.43	0.667

*N* = 154.

IM, Impression management; SDE, Self-deception enhancement; TSC, Trait self-control.

a = 0 = Alone, 1 = With other people.

We have conducted similar analyses involving SDE, TSC, and agreeableness. Their respective results are also summarized in Table 2. For SDE, the analysis uncovered significant effects for social context and SDE. However, there was no interaction. SDE was associated with reports of higher self-control while with other people, *b* = 0.47, *SE* = 0.06, 95% CI [0.34, 0.59], *t*(151) = 7.25, *p* < 0.001, η^2^_p_ = 0.258, and while alone, *b* = 0.45, *SE* = 0.07, 95% CI [0.32, 0.58], *t*(149) = 6.85, *p* < 0.001, η^2^_p_ = 0.239, to about the same extent.

For TSC, the analysis uncovered significant effects for social context and TSC, with no interaction effect. TSC was associated with reports of higher episodic self-control while with other people, *b* = 0.45, *SE* = 0.07, 95% CI [0.31, 0.58], *t*(152) = 6.58, *p* < 0.001, η^2^_p_ = 0.222, and while alone, *b* = 0.42, *SE* = 0.07, 95% CI [0.28, 0.56], *t*(149) = 6.02, *p* < 0.001, η^2^_p_ = 0.196, to about the same extent.^3^

The same pattern emerged for agreeableness. Significant effects were found for social context and agreeableness, but not for their interaction. Agreeableness was associated with reports of higher episodic self-control while with other people, *b* = 0.44, *SE* = 0.10, 95% CI [0.24, 0.65], *t*(151) = 4.30, *p* < 0.001, η^2^_p_ = 0.109, and while alone, *b* = 0.42, *SE* = 0.11, 95% CI [0.21, 0.63], *t*(150) = 3.99, *p* < 0.001, η^2^_p_ = 0.096, to about the same extent.

## Discussion

Concerns about social desirability are an integral part of psychological measurement, particularly in personality assessment (e.g., Barry et al., 2017; Müller and Moshagen, 2019; Williams et al., 2019). Scale development and application are often accompanied by control and correction for social desirability (e.g., MacCann et al., 2012; Maltby et al., 2019; Tse et al., 2020), and individuals scoring high on IM scales were considered to hold maladaptive qualities (e.g., Crowne, 1979).

However, the question of the validity of validity scales has yet to be resolved. Cumulative evidence indicates that these scales fail to measure faking, but, instead, tap personality substance (e.g., McCrae and Costa, 1983; Ones et al., 1996; Uziel, 2010b, 2014; De Vries et al., 2014; Zettler et al., 2015; Müller and Moshagen, 2019). However, much of this evidence builds on associations with broad personality constructs (e.g., McCrae and Costa, 1983; Müller and Moshagen, 2019). Recent works have begun to refine our understanding of the qualities that define IM, emphasizing sensitivity to social contexts and a response comprised of an increase in self-control (Uziel, 2010a, 2014). This approach has been supported in controlled experimental settings that manipulated the social context and explored the behavioral responses of individuals along the IM dimension (e.g., Kline et al., 2002; Uziel, 2010a; Uziel and Baumeister, 2012). However, evidence building on extended real-life dynamics in participants’ natural social environment was still lacking, as well as systematic comparisons between IM and related constructs.

To address these questions, the present research utilized an intensive longitudinal design (Bolger et al., 2003), whereby momentary levels of self-control were monitored more than 4,250 times (across 154 participants) alongside participants’ social condition. Results showed that, in general, people reported higher self-control in the presence of others (vs. alone) and that all four traits (IM, SDE, TSC, Agreeableness) were positively associated with episodic self-control. Addressing our main prediction, IM interacted with social condition, such that higher IM was associated with greater episodic self-control in the presence of other people than alone. High-IM individuals reacted to social presence with an increase in their self-control. In what follows, we discuss the implications of the findings.

The first effect that emerged was that in social presence (vs. alone), people reported greater self-control. This finding is consistent with existing studies using a similar experience sampling approach (Hofmann et al., 2012), and with laboratory-based experimental research (Erber et al., 1996). It reflects the notion that our behavior in social contexts is more controlled than our behavior alone because social presence motivates us to narrow discrepancies from desired standards (Carver and Scheier, 1981). Past (Uziel, 2007) and the present findings have shown that there are individual differences in how strong this effect is.

Turning to the main finding, past research has documented high self-control behavior in social contexts among high-IM individuals using behavioral measures (Blackhart et al., 2007; Uziel, 2010a; Uziel and Baumeister, 2012; Guo et al., 2019) and informant reports (Uziel, 2014). The present findings extend these findings to expressions of social behavior during everyday life. According to the IOSC model (Uziel, 2010b), individual differences in IM reflect a predisposition to react to social presence with an increase in self-control to maximize adaptation. High-IM individuals attribute importance to their social image but their behavior, personality correlates, and life outcomes indicate that their goal is not to impress others in deceitful or narcissistic manners but to integrate well into existing social structures (Uziel, 2010b; Bou Malham and Saucier, 2016). Exerting self-control in social contexts is central to the success of their approach because it assists in curbing the expression of impulses and adjusting behavior to external demands (Baumeister, 1982). Given that self-control resources are limited (Baumeister et al., 1998), it makes sense to conserve and apply them strategically when most needed (e.g., in public social contexts). Indeed, greater self-control in social contexts brings them desirable long-term payoffs by, for example, being considered trustworthy and conscientious (Paulhus, 1998; De Vries et al., 2014; Uziel, 2014).

Although the IOSC model is focused on adult personality, high-IM individuals’ behavioral profile (as expressed in the present study) shares commonalities with profiles described in developmental theories, such as in the ‘Adaptive Calibration Model’ (ACM)—an evolutionary-developmental theory of individual differences in the functioning of the stress system (Del Giudice et al., 2011). According to the ACM, adults’ high IM profile relates to a ‘sensitive pattern’ of responsivity, which develops in infancy in the context of attentive maternal care. This profile supports the development of an emotionally stable and socially competent adult personality, characterized by sensitivity to social feedback and the ability to mobilize metabolic and psychological resources when needed. Although more research is needed to study the developmental trajectories associated with IM, the similarity in repose patterns speaks to the potential adaptive benefits of this profile.

Responsiveness to social contexts (with an increase in self-control) was uniquely observed for IM as compared with the other traits measured. Although self-control is not the central component in the self-perception of individuals high on SDE (Paulhus, 1984), previous studies have found that high-SDE individuals report high self-control. However, informants did not corroborate these attributes (Uziel, 2014). Therefore, high-SDE individuals’ report of greater self-control (alone and in the presence of others to the same extent) is consistent with their self-deceiving bias and low sensitivity to social contexts (Paulhus, 1984; Uziel and Cohen, 2020).

TSC was also associated with greater self-control alone and with other people, as expected based on the definition of this trait (Tangney et al., 2004). Interestingly, recent approaches to TSC have suggested that high-TSC individuals do not necessarily exert more self-control on a momentary basis, because they strategically navigate their environment to reduce self-control conflicts (De Ridder et al., 2012; Hofmann et al., 2012). The present findings did not directly measure the presence of temptations or desires, but they indicate that across different social conditions, individuals high on TSC maintain a sense of having more self-control than individuals low on TSC. This could reflect their more efficient handling of conflicts overall, but it also implies that they do not calibrate their effort allocation to address conditions that require stronger responsiveness (Imhoff et al., 2014).

Agreeableness shares a modest correlation with IM (Graziano and Tobin, 2002), as was also found in the present data. However, the two constructs differ in the basic motives that account for the behavioral expressions associated with them. For the most part, Agreeableness is concerned with an emphatic concern for others’ welfare, whereas IM is focused on personal adjustment in social contexts. Notwithstanding, self-control is an important component in the development of agreeableness (Rothbart and Ahadi, 1994), and the present findings corroborate it at the trait and episodic levels. And yet, unlike IM, agreeableness was not differentially associated with levels of self-control alone and with other people. This fits our suggestion that greater self-control among individuals high on agreeableness is reserved for specific social settings, such as those involving emphatic behavior (Graziano et al., 2007).

The present study has several limitations. First, self-control was measured by self-reports and not by behavioral measures. This lends subjective value to the ratings, and thus limits to some extent their validity as in any study with self-reports. Notwithstanding, the design of the present study reduced its undesired impact because participants reported multiple times (> 27) while being in the comfort of their natural environment under terms of anonymity (vs. being observed in laboratory settings). Participants were therefore under little pressure to report in a socially desirable manner (Paulhus, 1984). Furthermore, the consistency of the present findings relating to IM with those found using alternative methods, including behavioral expressions of self-control (Uziel and Baumeister, 2012) is also reassuring that a systematic bias has little role in the association of IM with episodic self-control. An additional potential limitation concerns the generalizability of the sample. The present sample was comprised of educated young adults (mostly women) from a Western country. The theoretical claims about IM would benefit from expanding the sample composition to different populations varying in age and cultural values. Another limiting factor is the lack of specificity in the ‘with others’ context. Although this is a common approach in experience sampling research on self-control (Hofmann et al., 2012), it would have been informative to compare different compositions of social settings, especially by differentiating between settings involving greater and lesser self-presentational demands.

## Conclusion

In summary, the present research tested a substantive theory-driven meaning to individual differences in IM under natural conditions and differentiated IM from theoretically related constructs. Its conclusions are consistent with findings from controlled experiments in showing that IM predisposes individuals to respond to public social contexts with an increase in self-control, which may lead to an overall better adjustment. These findings carry practical implications concerning the use of IM scales as validity scales in assessment processes. IM may carry (substantive) adaptive rather than maladaptive qualities and removing variance associated with IM (or participants scoring high on IM scales) from analyses (or screening processes) is likely to reduce the quality of the measurement. The present findings are in line with and extend recent approaches (e.g., Ones et al., 1996; Uziel, 2010b, 2014; MacCann et al., 2012; De Vries et al., 2014; Connelly and Chang, 2016; Müller and Moshagen, 2019; Röhner et al., 2023) that call to reconsider the application of IM scales for these purposes and shift the focus to the substantive trait being measured.

## Data availablility statement

The datasets presented in this study can be found in online repositories. The names of the repository/repositories and accession number(s) can be found at: The Open Science Framework (OSF) at https://osf.io/kqy5t/.

## Ethics statement

This study involving humans was approved by Ethics Committee of the Department of Psychology, Bar-ilan University. The study was conducted in accordance with the local legislation and institutional requirements. The participants provided their written informed consent to participate in this study.

## Author contributions

LU and TSB: Study conceptualization, data collection, data analysis, report writing. TSB: data preparation. All authors contributed to the article and approved the submitted version.

## Funding

Preparation of this manuscript was supported by a grant from the Israel Science Foundation to LU (ISF grant No. 133/23).

## Conflict of interest

The authors declare that the research was conducted in the absence of any commercial or financial relationships that could be construed as a potential conflict of interest.

## Publisher’s note

All claims expressed in this article are solely those of the authors and do not necessarily represent those of their affiliated organizations, or those of the publisher, the editors and the reviewers. Any product that may be evaluated in this article, or claim that may be made by its manufacturer, is not guaranteed or endorsed by the publisher.

## Footnotes

^1^The dispute generally surrounds scales that tap onto Wiggins’s Gamma factor, such as the IM subscales of the BIDR (Paulhus, 1984), the MCSDS (Crowne and Marlowe, 1960) the Eysenck Personality Questionnaire-Revised (EPQ-R) Lie scale (Eysenck et al., 1985), and the MMPI Lie scale (Meehl and Hathaway, 1946; see Uziel, 2010b, for a review).

^2^The conclusions were unchanged by controlling for time (nor for sex or age).

^3^Because the items of the episodic self-control scale were based on (the long version of) the trait self-control scale (Tangney et al., 2004) there was some content overlap (in one item: “I am good at resisting temptation”) between our trait self-control scale (the brief trait self-control scale; Tangney et al., 2004) and our episodic self-control scale. To address complications that may arise from such an overlap, we recalculated the trait self-control score for this analysis omitting this item (i.e., retaining 12 items; α = 0.86). Results revealed negligible differences. Specifically, similar to the effects reported in Table 2, trait-self-control significantly predicted episodic self-control, *b* = 0.40, *SE* = 0.07,  *t*(149) = 5.74, *p* < 0.001, and there was no evidence for an interaction between trait self-control and social condition, *b* = 0.03, *SE* = 0.03,  *t*(148) = 0.81, *p* = 0.419.
